# Exploring the Ecology of Third Age Informal Language Learner Groups

**DOI:** 10.1093/geroni/igab046.3690

**Published:** 2021-12-17

**Authors:** Gareth Barnes

**Affiliations:** Macquarie University, Sydney, New South Wales, Australia

## Abstract

This research explores the factors involved in the emergence of an independently organized Third Age informal language learner group in a community centre in Japan. The methodology applies PPCT (Process-Person-Context-Time) from Bronfenbrenner’s bioecological approach to provide a detailed perspective of the people, the environment and settings over time to show how these factors interact to construct an emergent learner group. The analysis looks at how and why this specific learner ecology emerges and ultimately, how it can benefit the Third Age and inform healthy ageing policy. The findings show that by engaging in second language learning, the participants find meaningful and active involvement in the group by creating a setting that welcomes self-expression, while balancing limiting and facilitating factors of resilience and reciprocal support, self-management, sage-ing, interest, agency, and responsibility. The result is the creation of a multilingual, multicultural, and multigenerational place of inclusion within the community. The study highlights the heterogeneity of the 3rd Age and illustrates the interplay of contexts outside of the learner group from micro to macro, individual and group resources, and the influence of the specific social time period. It also shows the social importance of creating opportunities for autonomous informal language learning settings in the community while highlighting the impact of Third Age agency.

